# Cerebral Aneurysms and Arteriovenous Malformation: Preliminary Experience with the Use of Near-Infrared Fluorescence Imaging Applied to Endoscopy

**DOI:** 10.3390/jpm14121117

**Published:** 2024-11-22

**Authors:** Denis Aiudi, Alessio Iacoangeli, Andrea Mattioli, Alessio Raggi, Mauro Dobran, Gabriele Polonara, Riccardo Gigli, Maurizio Iacoangeli, Maurizio Gladi

**Affiliations:** 1 Department of Neurosurgery, Marche Polytechnic University, 60126 Ancona, Italy; andrea.mattioli.92@gmail.com (A.M.); raggi.alessio@gmail.com (A.R.); mauro.dobran@ospedaliriuniti.marche.it (M.D.); maurizio.iacoangeli@ospedaliriuniti.marche.it (M.I.); maurizio.gladi@ospedaliriuniti.marche.it (M.G.); 2 Department of Neuroradiology, Marche Polytechnic University, 60126 Ancona, Italy; gabriele.polonara@ospedaliriuniti.marche.it (G.P.); riccardo.gigli@ospedaliriuniti.marche.it (R.G.); 3 IRCCS INRCA, Marche Polytechnic University, 60124 Ancona, Italy

**Keywords:** indocyanine green video angiography, aneurysm, arteriovenous malformation, intraoperative angiography, intracranial Doppler, SPY mode endoscopy

## Abstract

**Background/Objectives:** Indocyanine green video angiography, integrated into the operative microscope, is frequently used in cerebrovascular surgery. This technology is often preferred, for cost or availability, to Doppler or intraoperative DSA (digital subtraction angiography). With the same assumption it was possible, in our preliminary experience, to partially vicariate the aforementioned devices using the SPY mode of the Stryker endoscope; it allowed the visualization of fluorescence in high definition. **Methods:** A retrospective analysis was conducted on a series of five patients suffering from cerebral aneurysm or AVM (arteriovenous malformation) who underwent, during the last year, surgical treatment with the aid of the microscope supported by the Stryker endoscope in the SPY mode for the visualization of the fluorescence emitted by indocyanine green. **Results:** All aneurysms were completely excluded from the cerebrovascular circulation in the absence of residues in the collar and occlusion of adjacent vessels; the complete removal of the nidus in all the AVMs was achieved with no residues. **Conclusions:** The intraoperative use of indocyanine green was a safe, rapid, and effective technique within a preliminary case study of “regular—not giant” aneurysms and superficially located AVM. The endoscopic technique in the SPY mode has allowed to partially vicariate the use of Doppler, intraoperative angiography, and integrated microscope video angiography. For these purposes, we propose, in selected cases, the support of the endoscope in the SPY mode during the microsurgical procedure in order to visualize the green fluorescence of indocyanine.

## 1. Introduction

Cerebral aneurysms and arteriovenous malformations (AVMs) are complex vascular pathologies of the brain that present significant risks of hemorrhage, stroke, and neurological deficits. Their intricate vascular anatomy, coupled with the critical nature of the brain’s blood supply, makes accurate diagnosis and effective surgical intervention crucial to patient outcomes.

Traditionally, intraoperative navigation and the visualization of these vascular anomalies rely on standard white-light endoscopy and advanced imaging modalities such as digital subtraction angiography (DSA) and magnetic resonance imaging (MRI). However, these techniques are limited by their inability to provide the real-time, high-resolution visualization of the cerebral vasculature during surgery. Microscopes and intraoperative technologies are constantly refined to improve outcomes and the complementary use of the indocyanine green video angiography (ICG-VA) with both endoscope and microscope has increased in recent decades, demonstrating efficacy and safety in cerebrovascular surgery; endoscope-integrated indocyanine is part of the advances in visualization tools with a great potential in visualizing vascular landmarks [1,2,3,4,5,6].

Near-infrared fluorescence (NIRF) imaging, an emerging technology, has demonstrated promise in enhancing the intraoperative visualization of vascular structures. By utilizing fluorescent dyes such as ICG that fluoresce under near-infrared light, NIRF imaging allows the real-time mapping of blood flow and vascular anatomy, providing superior contrast and specificity compared to traditional imaging techniques [7,8]. The SPY mode of the Stryker endoscope utilizes NIRF imaging to provide the real-time visualization of cerebral vasculature during surgery for cerebral aneurysms and AVMs. This technology works by detecting fluorescence from ICG dye injected into the bloodstream, allowing surgeons to visualize blood flow and identify vascular structures with high precision. The SPY mode is a display mode where a fluorescence image is shown in grayscale, providing the highest level of contrast between fluoresced and non-fluoresced [9,10,11,12,13,14].

ICG, an inert and water-soluble nonradioactive contrast agent that circulates only in the intravascular compartment, allows the real-time assessment and direct imaging of tissue perfusion and vascularization when enhanced by the laser in NIRF modality; fatal allergies and reactions are rare [9].

SPY mode offers an alternative, in selected cases and with a limited resolution, to traditional DSA or ICG video angiography, as it provides immediate, intraoperative feedback without the need for complex or expensive radiological setups. By enhancing vessel visualization, it aids in confirming aneurysm clipping or AVM resection, potentially reducing the dependence on angiography and improving surgical outcomes in a context where DSA or intraoperative ICG-VA is not available [10,11,12,13,14,15].

In this paper, we report our preliminary experience on the use of the SPY mode of the Stryker endoscope in cerebral aneurysms and AVM surgery. We aim to assess the benefits of this technique as a valid alternative to partially vicariate the aforementioned devices in improving the intraoperative identification of vascular structures, guiding surgical resection, and minimizing the risk of complications such as the incomplete obliteration of aneurysms or missing residual nidus in AVMs management. By evaluating the current literature and presenting case studies, we seek to demonstrate the potential of NIRF to enhance the safety and efficacy of neurovascular surgeries, offering a valuable tool in the neurosurgeon’s armamentarium.

## 2. Materials and Methods

### 2.1. Study Cohort and Surgical Equipment

ICG-enhanced visualization instruments can be integrated within surgical tools; in our series, we used a Leica M530 OHX microscope (Leica Microsystems, Wetzlar, Germany) without ICG filter and separately a Stryker 1688 AIM 4K endoscope (Kalamazoo, MI, USA) exploiting its SPY modality, able to illuminate the surgical field with a wavelength covering the ICG absorption band, to observe in real time angiographic images on the endoscope video screen after ICG intravenous injection (ICG is not reabsorbed into the intestine or hepatic circulation).

This retrospective observational study involved 5 patients: 3 cases of regular aneurysms and 2 cases of AVM located superficially in the cerebral parenchyma, who underwent surgery with the aid of the microscope supported by the Stryker endoscope in the SPY mode for the visualization of the fluorescence emitted by indocyanine green at our institution during 2023.

Patients’ data collection included clinical data and pre-/postoperative angiography and computed tomography angiography (CTA) images, together with intraoperative fluorescence images recorded using the endoscope. In each case, the surgical treatment of the vascular neurosurgical pathology was performed with the aid of the microscope supported by the Stryker endoscope in the SPY mode for the visualization of the fluorescence emitted by indocyanine green.

Ethical review and approval were waived for this study by Azienda Ospedaliero Universitaria delle Marche due to the retrospective nature of the study and being all the pro-cedures being part of the routine care.

### 2.2. Preparation Protocol

**Figure 1:**
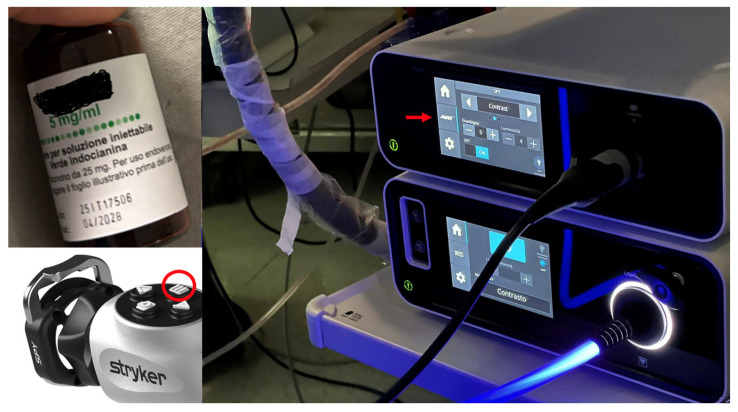
Example of the ICG available in our institution (**left**) and SPY mode activation button (red circle); real operative setting of the Stryker endoscope (**right**).

A protocol has been established in our institution that allows for collaboration between departments (this protocol is used in both AVM and aneurysm cases, with the same preparation, timing, and dosage instructions): ICG, especially in small institutions, is often not available in Neurosurgery departments but is common equipment in Ophthalmology or Hepato-Biliary Surgery Departments. ICG is not routinely available in our operating room, so in anticipation of the elective operation in which it will be used, it is made available specifically; the intravenous (iv) injection is made by mixing ICG with sterile water (its half-life is 3–5 min and elimination occurs within 15–20 min by the liver; maximum toxic dose is 5 mg/kg/day). The injection is performed a few seconds (5 to 15 s) before aiming the endoscope in the SPY mode at the affected and microsurgically treated vessel(s). The neurosurgical procedure is carried out mainly via a microscope and microsurgical standard techniques (i.e., clipping of the aneurysm or the microsurgical exclusion of the AVM nidus); since ICG, which is an inert and water-soluble nonradioactive contrast agent circulating only in the intravascular compartment, will not circulate in the presumably excluded AVM nidus or in the hypothetically clipped aneurysm sac if correctly treated, the “green vessels” will only be seen on the endosope screen in the normal surrounding parenchyma. The endoscope is used as a valid tool both for magnifying the anatomovascular structures and for confirming the correct success of the operation via the SPY mode function. The SPY mode of the Stryker endoscope, which allows for enhanced ICG circulating in the vessels, is activated through the following steps: selection, on the camera control unit of the endoscope tower, of the “laparoscopy mode”; and selection, on the camera control unit of the endoscope tower, of the “AIM (Advanced Image Modalities) logo” under the “home” icon (see the red arrow in Figure 1). Once in SPY mode, we go ahead with selection, again on the camera control unit of the endoscope tower, of the “contrast modality” by clicking on the arrows on the screen. At this point, the SPY mode is by default in grayscale on the screen, and therefore, to enhance the green color modality, the operator from the sterile field will need to press the button on the right of the sterile camera before aiming with the optic to the treated vessel(s) (there are 4 buttons on the camera head, press the one on the right to turn on/off the SPY mode; see red circle in Figure 1).

### 2.3. Use of the Endoscope with Fluorescence Visualization and IntraOperative Evaluation

After the clipping of the aneurysm or microsurgical exclusion of the AVM nidus, a single 25 mg bolus of ICG was injected by iv route. The endoscope in the SPY mode for the visualization of fluorescence was positioned in the surgical field to visualize the vascular structures stained by the intravascular passage of the ICG. The angiographic images can be observed in real time and recorded on the video screen approximately 15 s after the injection. In patients with cerebral aneurysms, the endoscope and fluorescence made it possible to evaluate the patency of the vessel from which the aneurysm originates and of the surrounding ones, together with the absence of residual neck near the clip, also through the use of 30- and 45-degree-angled optics. In cases of AVM removal, endoscopic visualization was used to analyze the absence of lesional residues by exploring the cavity walls. No adverse reactions were observed following ICG infusion.

## 3. Results

### 3.1. Patients Overview

**Figure 2:**
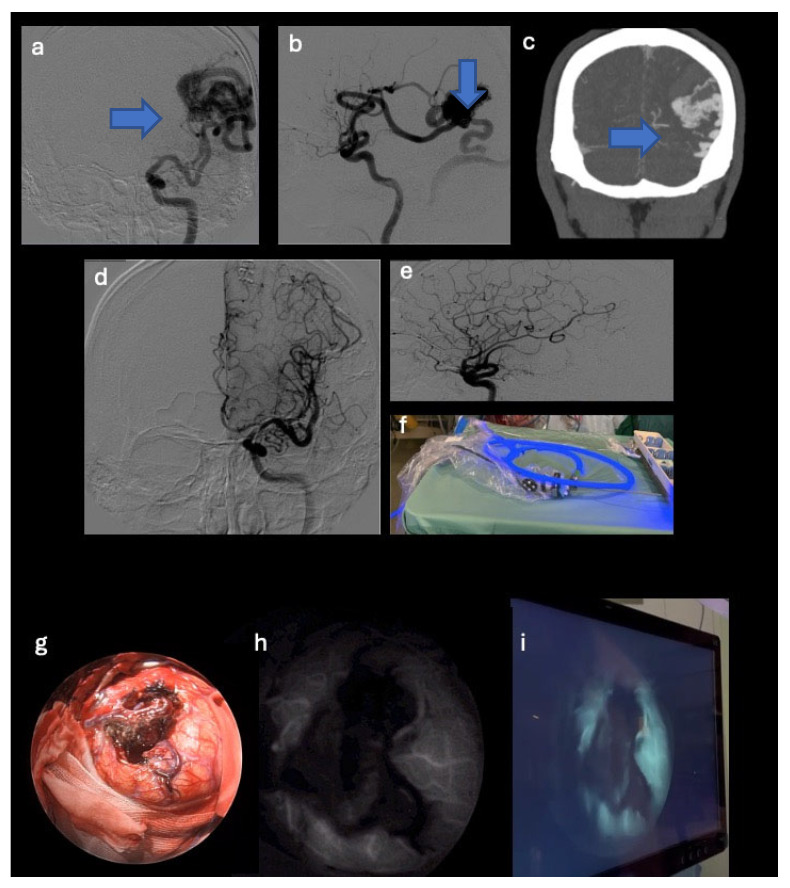
Preoperative digital subtraction angiography and computed tomography angiography images that show an arteriovenous malformation (blue arrows) originating from the left middle cerebral artery and venous drainage into the ipsilateral transverse sinus (**a**–**c**). Postoperative digital subtraction angiography images that highlight the exclusion of the nidus (**d**,**e**). Endoscope equipment (**f**), standard endoscopic view (**g**), and endoscope in the SPY mode ready for intraoperative use demonstrating the arteriovenous malformation exclusion (**h**,**i**).

The five patients who underwent surgery had the following clinical and neuroradiological characteristics:

(1) A 57-year-old male patient with a middle cerebral artery aneurysm (M2 segment) with a diameter of 1.2 cm, neurologically intact. The patient underwent clipping of the aneurysm neck.
(2) A 73-year-old female patient with temporal AVM originating from the left middle cerebral artery and discharge into the ipsilateral transverse sinus, with a 3 cm nidus. The AVM manifested itself with an episode of fluent aphasia. The patient underwent preoperative embolization and microsurgical removal of the nidus (see Figure 1).
(3) A 46-year-old female patient with an aneurysm of the anterior communicating artery with a diameter of 1 cm, neurologically intact. The patient underwent clipping of the aneurysm neck.
(4) A 44-year-old female patient with a right middle cerebral artery aneurysm (M1-M2 union) with a diameter of 1 cm, a history of headaches. The patient underwent clipping of the aneurysm neck (see Figure 2).
(5) A 31-year-old male patient with frontal AVM originating from the right anterior cerebral artery and discharge into the sagittal sinus, with a 2.5 cm nidus. The patient presented with weakness in the left upper limb and underwent microsurgical removal of the nidus.

The intraoperative use of indocyanine green was a safe, rapid, and effective technique, and no adverse reactions occurred; the five patients who underwent surgery had the following clinical and neuroradiological characteristics summarized in Table 1 and in the Illustrative Cases Section.

### 3.2. Illustrative Cases

#### 3.2.1. Case Number 2

The illustrative case number 2 demostrates the possibility to verify AVM exclusion through the SPY mode modality (see Figure 2).

#### 3.2.2. CASE Number 4

**Figure 3:**
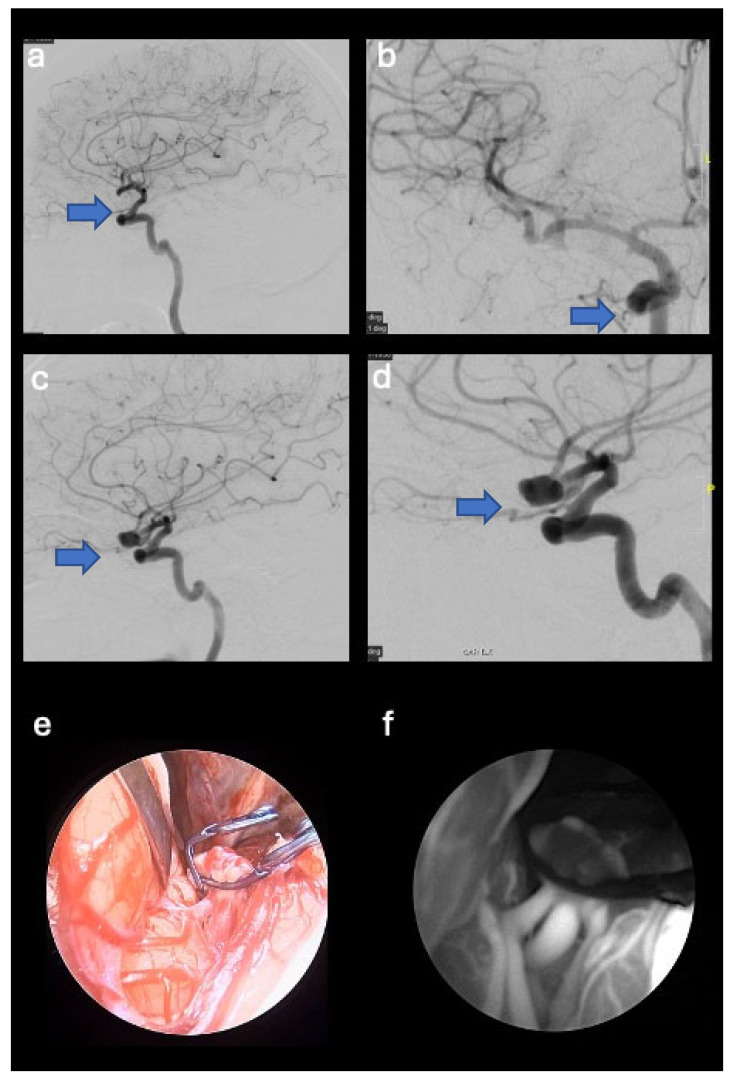
Pre- and postoperative digital subtraction angiography images that show right multilobed middle cerebral artery aneurysm (blue arrows) (M1-M2 union) (**a**,**b**). Postoperative digital subtraction angiography image of the excluded aneurysm (**c**,**d**). Standard endoscopic view (**e**) and endoscope in the SPY mode (**f**).

The illustrative case number 4 demostrates the possibility to verify aneurysm exclusion through the SPY mode modality (see Figure 3).

## 4. Discussion

ICG is a water-soluble dye; the first application in the medical field dates to 1956 for the study of cardiovascular and liver function, while in 1970, its fluorescence properties were exploited in the ophthalmological field; the advantage of ICG over fluorescein is that the light emission is more intense and easier to detect, and the adverse reactions are also very low. ICG was approved by the US Food and Drug Administration in 1956 and 1975 for cardiocirculatory measurements, liver function tests, and ophthalmic angiography. Only in 2003 it was introduced into the neurosurgical practice to perform angiographic studies, and it is frequently used in cerebrovascular surgery; the applications of ICG have expanded rapidly across different specialties since its initial development [7,8,9,10,11,12]. Teng et al. described the application of ICG in neuro-oncology from gliomas and not only to pituitary adenomas [13,14,15,16,17,18]; Mansour et al. reported its application in clipping a ruptured anterior spinal artery (ASA) aneurysm [8].

Endoscopy in our series was extremely useful because of its simple and reliable method for acquiring high spatial resolution images in real time. In addition, in a context where intraoperative angiography and operating microscope with ICG filter are not available, because of its large diffusion, endoscopy can potentially reduce costs and be much more available than the abovementioned intraoperative tools [18,19,20,21,22,23,24]; it can be improved combining Doppler and can potentially overcome the dead angles of the microscope as reported by Hashimoto et al. [25]. Catapano et al. described a 40-patient endoscope-integrated ICG fluorescence case series with no perioperative complications, reporting endoscopy as a potentially critical tool in endonasal, intraventricular, aneurysm and brain tumor surgeries; furthermore, the same authors observed that the fluorescence observed with the endoscope was more prolonged than that visible with the microscope [26]. Wong et al. demonstrated the possible application of endoscopic ICG video angiography in challenging posterior circulation aneurysm cases [27], while in anterior circulation ones, Chen et al. reported a potentially reduced cerebral vasospasm combining neuroendoscopy with ICG because of the decreased risk of misclipping perforating branches [28,29,30,31,32,33,34,35,36].

Despite the abovementioned promising features, especially in contexts where intraoperative video angiography is not available, endoscopy has its own limitations and intraoperative DSA remains the gold standard technique in cerebrovascular surgery (especially with deep-seated AVMs or when managing thick-walled atherosclerotic vessels).

The main purpose of aneurysm surgery is to completely exclude them from the cerebral vascular circulation in the absence of residues in the collar and occlusion of adjacent vessels; similarly, in AVM surgery, the goal is the early identification of the nidus and to avoid residues within it given the high risk of new rupture. In our brief clinical experience presented, the endoscope allowed the excellent visualization of the vascular structures and the meticulous exploration of the bottom and walls of the nidus of the removed AVM, sometimes covered by the overlying parenchyma, which the microscope would not have allowed. Furthermore, the use of angled optics allows blind spots to be reached without the manipulation of the surrounding nervous tissue.

ICG-VA is often preferred, for cost or availability, to Doppler or intraoperative angiography DSA, and with the same assumption it was possible, in our preliminary experience, to partially vicariate the aforementioned devices using the SPY mode of the Stryker endoscope allowing the visualization of fluorescence in high definition.

According to the literature, the intraoperative use of ICG was a safe, rapid, and effective technique within a preliminary case study of “regular—not giant” aneurysms and AVM superficially located in the cerebral parenchyma.

In our preliminary experience, the use of the ICG combined with the SPY mode of the Stryker endoscope has provided adequate information regarding the correct clipping of aneurysms and the early identification of AVM nidus and potentially residues; in specific contexts where gold standard technologies are not available, this technology can be a valuable tool in the complete removal of the nidus and exclusion of aneurysms: in our preliminary experience, a satisfying management of the disease, a clinical improvement in, and the absence of bleeding events were observed in all the cases.

Limitations are present as previously mentioned and future research directions could overcome the limits of both microscope and endoscope with new technologies as suggested by Ferlendis et al. who proposed ultrahigh-definition 3-dimensional exoscopes that combine the advantages of operating microscopes and endoscopes. New technological innovations offer optimal lighting and magnification considering ergonomics as well [5]. Research and new surgical tools such as exoscopes or highly technologically integrated microscopes could take time to be widespread, so our group suggests that the use of ICG combined with the endoscopic technique in the SPY mode could partially vicariate, in selected cases, the use of intraoperative angiography and integrated microscope video angiography where they are not available.

## 5. Conclusions

In circumstances where intraoperative angiography is not available, in order to verify aneurysms’ exclusion and to identify AVM residual nidus early, we propose, in selected cases and keeping in mind the described limitations of the technique that are principally deep-seated AVMs and thick-walled atherosclerotic vessels in complex aneurysm management, the support of the endoscope in SPY mode during the microsurgical procedure in order to enhance and visualize the green fluorescence of indocyanine; the described protocol is used in both AVM and aneurysm cases and can be useful to be reproduced for colleagues working in a similar context. Although the procedure is carried out mainly via a microscope, the endoscope proves to be a valid tool both for magnifying the anatomovascular structures and for confirming the correct success of the operation via the SPY mode function.

## Figures and Tables

**Table 1:** Clinical, pre-, and postoperative data of patients who underwent surgery. Abbreviations. M, male; F, female.

	Age	Sex	Clinical Data	CV Disease	Neurosurgical Procedure	Postoperative Outcome
1	57	M	Neurologically intact	Left middle cerebral artery aneurysm (M2 segment) with a diameter of 1.2 cm	Clipping of the aneurysm neck	Neurologically intact; total exclusion of the aneurysm. No bleeding events.
2	73	F	Episode of fluent aphasia	Left temporal AVM originating from the left middle cerebral artery and venous drainage into the ipsilateral transverse sinus (3 cm nidus)	Preoperative embolization and the microsurgical removal of the nidus	Complete removal of the nidus and transient fluent aphasia lasting three months; no bleeding events.
3	46	F	Neurologically intact	Anterior communicating artery aneurysm with a diameter of 1 cm	Clipping of the aneurysm neck	Neurologically intact, total exclusion of the aneurysm, and no bleeding events.
4	44	F	Headache	Right middle cerebral artery aneurysm (M1-M2 union) with a diameter of 1.1 cm	Clipping of the aneurysm neck	Neurologically intact, total exclusion of the aneurysm, and no bleeding events.
5	31	M	Weakness in the left upper limb	Right frontal AVM originating from the right anterior cerebral artery and venous drainage into the sagittal sinus (2.5 cm nidus)	Microsurgical removal of the nidus	Complete removal of the nidus and partial recovery of weakness. No bleeding events.

## Data Availability

Data is unavailable due to privacy and ethical restrictions.

## References

[1] De Notaris M., Sacco M., Corrivetti F., Dallan I., Cavallo L.M., Somma T., Parbonetti G., Colamaria A., Solari D. (2022). Indocyanine Green Endoscopy for Pituitary Adenomas with Parasellar Extension: Results from a Preliminary Case Series. World Neurosurg..

[2] Srinivasan V.M., Shlobin N.A., Karahalios K., Scherschinski L., Rahmani R., Graffeo C.S., Burkhardt J.K., Chaurasia B., Catapano J.S., Labib M.A. (2022). Adoption of Advanced Microneurosurgical Technologies: An International Survey. World Neurosurg..

[3] Vega-Moreno D.A., Janković D., Azouz H., Nakipuria M., Kato Y. (2023). Dual Microscope Indocyanine Green Video Angiography and Endoscopic Review to Treat Intracranial Aneurysm: A Review of the Literature Regarding a Case. Asian J. Neurosurg..

[4] Muto J., Mine Y., Nishiyama Y., Murayama K., Hayakawa M., Hasegawa M., Lee J.K.Y., Hirose Y. (2023). Intraoperative Real-Time Near-Infrared Image-Guided Endoscopic Endonasal Surgery for Pituitary Tumors. World Neurosurg..

[5] Ferlendis L., Veiceschi P., Capelli S., Agresta G., Leocata A., Pozzi F., Locatelli D. (2023). Ultrahigh-Definition-3-Dimensional Exoscope-Assisted Clipping of a Right Middle Cerebral Artery Unruptured Aneurysm with Indocyanine Green Video Angiography: Operative Video. World Neurosurg..

[6] Inokuchi G., Mine M., Tamagawa K., Tatehara S., Yui M., Uozumi Y., Fujita Y., Nakai T., Nibu K.I. (2024). Indocyanine green fluorescence visualizes landmark arteries for endoscopic sinus and skull base surgery. Am. J. Otolaryngol..

[7] Della Puppa A., Rossetto M., Volpin F., Rustemi O., Grego A., Gerardi A., Ortolan R., Causin F., Munari M., Scienza R. (2018). Microsurgical Clipping of Intracranial Aneurysms Assisted by Neurophysiological Monitoring, Microvascular Flow Probe, and ICG-VA: Outcomes and Intraoperative Data on a Multimodal Strategy. World Neurosurg..

[8] Mansour A., Endo T., Inoue T., Sato K., Endo H., Fujimura M., Tominaga T. (2019). Clipping of an anterior spinal artery aneurysm using an endoscopic fluorescence imaging system for craniocervical junction epidural arteriovenous fistula: Technical note. J. Neurosurg. Spine.

[9] Devgan Y., Mayilvaganan S., Mishra A., Chand G., Agarwal G., Agarwal A. (2024). Comparison of indocyanine green angiography vs. intraoperative parathyroid hormone in early prediction of risk of post-thyroidectomy hypocalcemia: A prospective cohort study. Ann. Med. Surg..

[10] Teng C.W., Huang V., Arguelles G.R., Zhou C., Cho S.S., Harmsen S., Lee J.Y.K. (2021). Applications of indocyanine green in brain tumor surgery: Review of clinical evidence and emerging technologies. Neurosurg. Focus.

[11] Ferroli P., Acerbi F., Albanese E., Tringali G., Broggi M., Franzini A., Broggi G. (2011). Application of intraoperative indocyanine green angiography for CNS tumors: Results on the first 100 cases. Intraoperative Imaging.

[12] Cho S.S., Salinas R., Lee J.Y.K. (2019). Indocyanine-Green for Fluorescence-Guided Surgery of Brain Tumors: Evidence, Techniques, and Practical Experience. Front. Surg..

[13] Yannuzzi L.A. (2011). Indocyanine green angiography: A perspective on use in the clinical setting. Am. J. Ophthalmol..

[14] Lau C.T., Au D.M., Wong K.K.Y. (2019). Application of indocyanine green in pediatric surgery. Pediatr. Surg. Int..

[15] Raabe A., Beck J., Gerlach R., Zimmermann M., Seifert V. (2003). Near-infrared indocyanine green video angiography: A new method for intraoperative assessment of vascular flow. Neurosurgery.

[16] Jeon J.W., Cho S.S., Nag S., Buch L., Pierce J., Su Y.S., Adappa N.D., Palmer J.N., Newman J.G., Singhal S. (2019). Near-Infrared Optical Contrast of Skull Base Tumors During Endoscopic Endonasal Surgery. Oper Neurosurg.

[17] Della Puppa A., Rustemi O., Scienza R. (2017). The “ICG Entrapment Sign” in Cerebral Aneurysm Surgery Assisted by Indocyanine Green Videoangiography. World Neurosurg..

[18] Balamurugan S., Agrawal A., Kato Y., Sano H. (2011). Intra operative indocyanine green video-angiography in cerebrovascular surgery: An overview with review of literature. Asian J. Neurosurg..

[19] Chibbaro S., Tacconi L. (2006). Extracranial-intracranial bypass for the treatment of cavernous sinus aneurysms. J. Clin. Neurosci..

[20] Takagi Y., Samamura K., Hashimoto N. (2008). Intra operative Near-infrared Indocyanine green video angiography performed with surgical microscope—Applications in Cerebrovascular surgery. Eur. Neurol. Rev..

[21] Mery F.J., Amin-Hanjani S., Charbel F.T. (2008). Is an angiographically obliterated aneurysm always secure?. Neurosurgery.

[22] Della Puppa A., Rustemi O., Rossetto M., Gioffrè G., Munari M., Charbel F.T., Scienza R. (2014). The “squeezing maneuver” in microsurgical clipping of intracranial aneurysms assisted by indocyanine green videoangiography. Neurosurgery.

[23] Zitek H., Hejcl A., Sadeh M., Charbel F.T., Sames M. (2024). Occipital artery to vertebral artery bypass for treatment of bilateral vertebral artery occlusion with QMRA as an adjunct to diagnostic assessment. Acta Neurochir.

[24] Acerbi F., Prada F., Vetrano I.G., Falco J., Faragò G., Ferroli P., DiMeco F. (2019). Indocyanine Green and Contrast-Enhanced Ultrasound Videoangiography: A Synergistic Approach for Real-Time Verification of Distal Revascularization and Aneurysm Occlusion in a Complex Distal Middle Cerebral Artery Aneurysm. World Neurosurg..

[25] Hashimoto K., Kinouchi H., Yoshioka H., Kanemaru K., Ogiwara M., Yagi T., Wakai T., Fukumoto Y. (2017). Efficacy of Endoscopic Fluorescein Video Angiography in Aneurysm Surgery-Novel and Innovative Assessment of Vascular Blood Flow in the Dead Angles of the Microscope. Oper Neurosurg.

[26] Catapano G., Sgulò F., Laleva L., Columbano L., Dallan I., de Notaris M. (2018). Multimodal use of indocyanine green endoscopy in neurosurgery: A single-center experience and review of the literature. Neurosurg. Rev..

[27] Wong A.K., Wong R.H. (2020). Keyhole clipping of a low-lying basilar apex aneurysm without posterior clinoidectomy utilizing endoscopic indocyanine green video angiography. Surg. Neurol. Int..

[28] Bruneau M., Appelboom G., Rynkowski M., Van Cutsem N., Mine B., De Witte O. (2013). Endoscope-integrated ICG technology: First application during intracranial aneurysm surgery. Neurosurg. Rev..

[29] De Oliveira J.G., Beck J., Seifert V., Teixeira M.J., Raabe A. (2008). Assessment of flow in perforating arteries during intracranial aneurysm surgery using intraoperative near-infrared indocyanine green videoangiography. Neurosurgery.

[30] Fischer G., Rediker J., Oertel J. (2018). Endoscope-versus microscope-integrated near-infrared indocyanine green videoangiography in aneurysm surgery. J. Neurosurg..

[31] Kalavakonda C., Sekhar L.N., Ramachandran P., Hechl P. (2002). Endoscope-assisted microsurgery for intracranial aneurysms. Neurosurgery.

[32] Mielke D., Malinova V., Rohde V. (2014). Comparison of intraoperative microscopic and endoscopic ICG angiography in aneurysm surgery. Neurosurgery.

[33] Nishiyama Y., Kinouchi H., Senbokuya N., Kato T., Kanemaru K., Yoshioka H., Horikoshi T. (2012). Endoscopic indocyanine green video angiography in aneurysm surgery: An innovative method for intraoperative assessment of blood flow in vasculature hidden from microscopic view. J. Neurosurg..

[34] Washington C.W., Zipfel G.J., Chicoine M.R., Derdeyn C.P., Rich K.M., Moran C.J., Cross D.T., Dacey R.G. (2013). Comparing indocyanine green videoangiography to the gold standard of intraoperative digital subtraction angiography used in aneurysm surgery. J. Neurosurg..

[35] Oda J., Kato Y., Chen S.F., Sodhiya P., Watabe T., Imizu S., Sano H., Hirose Y. (2011). Intraoperative near-infrared indocyanine green-videoangiog-raphy (ICG-VA) and graphic analysis of fluorescence intensity in cerebral aneurysm surgery. J. Clin. Neurosci..

[36] Chen D.Y., Xu C.S., Fu K., Ma Y.H., Zhang T.B., Zou Y.C., Chen J.C. (2021). Application of neuroendoscopy combined with fluorescence angiography in anterior circulation aneurysm clipping. Zhonghua Yi Xue Za Zhi.

